# The platelet transcriptome and proteome in Alzheimer’s disease and aging: an exploratory cross-sectional study

**DOI:** 10.3389/fmolb.2023.1196083

**Published:** 2023-06-30

**Authors:** Diana M. Bessa de Sousa, Rodolphe Poupardin, Saul A. Villeda, Adam B. Schroer, Thomas Fröhlich, Vanessa Frey, Wolfgang Staffen, Heike Mrowetz, Barbara Altendorfer, Michael S. Unger, Bernhard Iglseder, Bernhard Paulweber, Eugen Trinka, Janne Cadamuro, Martin Drerup, Katharina Schallmoser, Ludwig Aigner, Kathrin M. Kniewallner

**Affiliations:** ^1^ Institute of Molecular Regenerative Medicine, Paracelsus Medical University, Salzburg, Austria; ^2^ Spinal Cord Injury and Tissue Regeneration Center Salzburg (SCI-TReCS), Paracelsus Medical University, Salzburg, Austria; ^3^ Experimental and Clinical Cell Therapy Institute, Paracelsus Medical University, Salzburg, Austria; ^4^ Department of Anatomy, University of California San Francisco, San Francisco, CA, United States; ^5^ Laboratory of Functional Genome Analysis (LAFUGA), Gene Center, Ludwig Maximilian University of Munich, Munich, Germany; ^6^ Department of Neurology, Christian Doppler Clinic, Paracelsus Medical University, Salzburg, Austria; ^7^ Department of Internal Medicine, St. Johanns University Hospital, Paracelsus Medical University, Salzburg, Austria; ^8^ Department of Public Health, Health Services Research and Health Technology Assessment, UMIT-University for Health Sciences, Medical Informatics and Technology, Hall in Tirol, Austria; ^9^ Neuroscience Institute, Christian Doppler University Hospital, Paracelsus Medical University and Centre for Cognitive Neuroscience Salzburg, Salzburg, Austria; ^10^ Department of Laboratory Medicine, University Hospital SALK, Salzburg, Austria; ^11^ Department of Urology, Paracelsus Medical University, Salzburg, Austria; ^12^ Department of Transfusion Medicine, Paracelsus Medical University, Salzburg, Austria; ^13^ Austrian Cluster for Tissue Regeneration, Vienna, Austria

**Keywords:** Alzheimer’s disease, aging, platelets, proteomics, transcriptomics

## Abstract

**Introduction:** Alzheimer’s disease (AD) and aging are associated with platelet hyperactivity. However, the mechanisms underlying abnormal platelet function in AD and aging are yet poorly understood.

**Methods:** To explore the molecular profile of AD and aged platelets, we investigated platelet activation (i.e., CD62P expression), proteome and transcriptome in AD patients, non-demented elderly, and young individuals as controls.

**Results:** AD, aged and young individuals showed similar levels of platelet activation based on CD62P expression. However, AD and aged individuals had a proteomic signature suggestive of increased platelet activation compared with young controls. Transcriptomic profiling suggested the dysregulation of proteolytic machinery involved in regulating platelet function, particularly the ubiquitin-proteasome system in AD and autophagy in aging. The functional implication of these transcriptomic alterations remains unclear and requires further investigation.

**Discussion:** Our data strengthen the evidence of enhanced platelet activation in aging and provide a first glimpse of the platelet transcriptomic changes occurring in AD.

## 1 Introduction

Alzheimer’s disease (AD) is the most common form of dementia among elderly individuals. It is a complex pathology mainly characterized by the presence of misfolded protein aggregates in the brain (i.e., amyloid plaques and neurofibrillary tau tangles), neuroinflammation, neuronal death and cerebrovascular dysfunction (Cortes-Canteli and Iadecola, 2020). Aging is the major risk factor for AD, and age-associated vascular dysfunction and chronic inflammation seem to strongly link both conditions (Xia et al., 2018).

Platelets are key effector blood cells in hemostasis, inflammation, and immune response (Davizon-Castillo et al., 2019; Le Blanc and Lordkipanidzé, 2019; Montenont et al., 2019). Platelets circulate in the blood in a resting status, becoming activated in response to noxious stimuli such as vascular damage and inflammation. Platelet activation is a complex process, generally characterized by the upregulation of surface molecules, cytoskeletal remodeling, and granule content secretion, that allows platelets to execute their functions.

Aging (Davizon-Castillo et al., 2019; Le Blanc and Lordkipanidzé, 2019) and AD (Sevush et al., 1998; Ciabattoni et al., 2007) are associated with enhanced platelet activation. While in aging, chronic systemic inflammation, particularly to elevated plasma levels of TNF-alpha, was recently linked to platelet hyperactivity (Davizon-Castillo et al., 2019), in AD, amyloid beta peptides (Abeta) might drive enhanced platelet activation (Shen et al., 2008; Canobbio et al., 2014; Sonkar et al., 2014; Donner et al., 2016). However, the molecular mechanisms underlying an abnormal platelet function in aging and AD remain largely unexplored.

Platelets inherit most of their protein and RNA content from megakaryocytes, but their proteomic and transcriptomic profiles dynamically change in response to different conditions, such as aging, infection or cancer (Simon et al., 2014; Caparrós-Pérez et al., 2017; Campbell et al., 2018; Davizon-Castillo et al., 2019). AD platelets significantly differ from aged platelets in the expression of several proteins (Zellner et al., 2012; Donovan et al., 2013; González-Sánchez et al., 2018; Reumiller et al., 2018; Yu et al., 2021), microRNAs (Lee et al., 2020; Gámez-Valero et al., 2021) and metabolites (Oberacher et al., 2017), and many of these molecular alterations might serve as diagnostic biomarkers (Veitinger et al., 2014; Oberacher et al., 2017; Akingbade et al., 2018; Yu et al., 2021). Here, we used LC-MS/MS proteomics and mRNAseq to analyze the proteome and transcriptome of platelets from AD, aged non-demented (Old) and young (Young) individuals, aiming to explore whether AD and aging modulate the molecular profile of platelets differentially and gain further insight into the mechanisms supporting platelet activation in AD and aging.

## 2 Materials and methods

### 2.1 Participant selection

A total of 39 individuals participated in this study, including 11 probable AD patients, 18 non-demented elderly (Old), and 10 young control individuals (Young). Study participants were recruited at the University Hospital Salzburg, Austria and at the Paracelsus Medical University, Salzburg, Austria. The cognitive assessment was performed using the Mini-Mental State Examination (MMSE) in AD patients, and using the Salzburg Dementia Test Prediction (SDTP), a three-question cognitive screening tool that predicts MMSE scores, in Old controls (Kaiser et al., 2014). The cognitive fitness of young participants was not assessed. The exclusion criteria included the intake of medication that interferes with platelet function, as specified in Supplementary Table S1. All participants signed an informed consent form approved by the local ethics committee (415-E/2311/33-2020). The demographic characteristics of the study population are presented in Table 1.

**TABLE 1:** Demographic characteristics of the study population.

	AD	Old	Young	*p*-value
Platelet activation	*n* = 11	*n* = 18	*n* = 10	
Sex female, *n* (%)	7 (64%)	9 (50%)	3 (30%)	
Age range, years	61–83	64–76	18–25	
Age, years, mean (SD)	74.4 (7.2)	69.5 (2.6)	20.8 (2.1)	**0.0327**
MMSE^a^, mean (SD)	21.7 (4.0)	28.0 (0.0)	n.d.	**<0.0001**
Proteomics	*n* = 7	*n* = 7	*n* = 9	
Sex female, *n* (%)	5 (71%)	3 (43%)	2 (22%)	
Age range, years	61–80	70–76	18–25	
Age, years, mean (SD)	73.1 (7.5)	71.4 (2.1)	20.9 (2.3)	0.2413
MMSE^a^, mean (SD)	20.0 (4.8)	28.0 (0.0)	n.d.	**0.0010**
Transcriptomics	*n* = 9	*n* = 9	*n* = 9	
Sex female, *n* (%)	5 (56%)	5 (56%)	2 (22%)	
Age range, years	61–83	64–76	18–25	
Age, years, mean (SD)	74.2 (7.8)	70.4 (2.6)	21.0 (2.2)	0.1776
MMSE^a^, mean (SD)	20.8 (4.2)	28.0 (0.0)	n.d.	**0.0002**

^a^ Mini-Mental State Examination (MMSE). In Old controls, MMSE, score was predicted using the Salzburg Dementia Test Prediction (SDTP). *P*-value indicates statistical differences between AD, and Old groups (age: Mann-Whitney test; MMSE: unpaired *t*-test). Significant *p*-values (<0.05) are highlighted in bold.

### 2.2 Platelet isolation from whole blood

**FIGURE 1:**
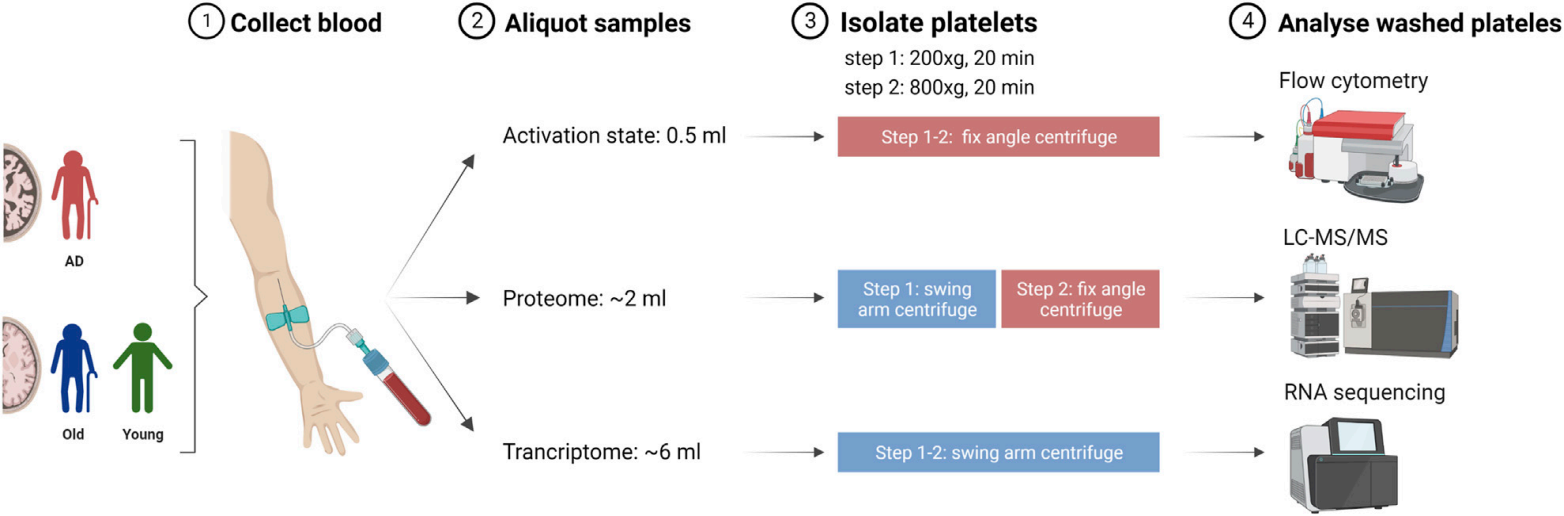
Schematic representation of blood collection and processing for platelet isolation and analysis. Blood collected by peripheral venipuncture from Alzheimer’s disease (AD) patients, non-demented elderly (Old) and young individuals (Young) was aliquoted and processed for platelet isolation using a two-step centrifugation approach. Created with Biorender.

Blood sampling was performed at the University Hospital Salzburg by trained medical staff. Blood was collected by peripheral venipuncture into Vacuette^®^ 9NC coagulation sodium citrate 3.2% blood collection tubes. Blood samples were aliquoted and processed for platelet isolation, as described in Figure 1. Blood samples were centrifuged at 200 × g for 20 min, RT to collect platelet rich plasma. For transcriptome and proteome analysis, the upper two-thirds of platelet rich plasma (PRP) fraction were transferred into a new tube. For platelet activation, all PRP was collected. Prostaglandin E1 (1 µM) was added to minimize artificial platelet activation, and samples were centrifuged at 800 × g for 20 min, RT. Platelet pellets were washed twice with Tyrode’s buffer (134 mM NaCl, 12 mM NaHCO3, 2.9 mM KCl, 1 mM MgCl2, 0.34 mM Na2HPO4, 10 mM Hepes solution). For platelet activation analysis, platelets were resuspended in FC buffer [2% bovine serum albumin (Sigma), 2 mM ethylenediaminetetraacetic acid (EDTA, Promega) in PBS Dulbecco (Merck)] and processed for flow cytometry. For transcriptomics, platelets were resuspended in RNAlater^®^ (Sigma) and stored at −80°C. For proteomic analysis, platelet pellets were immediately stored at −80°C.

### 2.3 Platelet activation

#### 2.3.1 Flow cytometry

We assessed platelet activation using two-color flow cytometry in 11 AD patients, 18 Old and 10 Young controls. Washed platelets (100 µL) were incubated with FC buffer (100 µL) containing FC pre-block solution (1:20; # 564220, BD Biosciences) for 10 min at RT to block unspecific antibody binding. For antibody staining, mouse anti-human fluorescein isothiocyanate (FITC)-labelled CD41a antibody (1:20; #561851; BD Biosciences) and mouse anti-human allophycocyanin-(APC) CD62P labelled antibody (1:20; #561920; BD Biosciences) were added. As a control for CD62P expression, isotype IgG staining was performed using mouse anti-human APC-IgG1k isotype (1:20; #550854; BD Biosciences). After 30 min incubation in the dark at RT, the staining was stopped by dilution with FC buffer, and samples were centrifuged for 5 min at 800 × g. Platelet pellets were resuspended in FC buffer and immediately analyzed with BD Accuri TM C6 Plus Flow Cytometer (BD Biosciences).

Platelet population was defined based on their forward and side scattering pattern on a logarithmic scale and positive staining for CD41, a commonly used pan-platelet marker. Cell doublets were excluded from the analysis. To assess platelet activation, CD41^+^ cells were gated for CD62P^+^ expression. CD62P (also known as P-selectin) is a platelet α-granule protein only expressed at the platelet surface upon activation (Michelson et al., 2005).

#### 2.3.2 Statistically analysis

Statistical analysis was performed using GraphPad Prism (Version 7.00). Data were tested for normality using the Shapiro-Wilk normality test, and statistical analysis between groups was performed by one-way ANOVA with Holm-Šídák’s multiple comparisons test. Correlation analysis between platelet activation and MMSE score in AD individuals was performed by simple linear regression. Data are depicted as mean ± standard error of the mean (SEM).

### 2.4 Proteomic analysis–Mass spectrometry

Platelet samples were lysed in 10 µL 8 M Urea/0.4 M NH4HCO3 and sonicated with 11 pulses of 10 s each using a Sonopuls HD3200 (Bandelin, Berlin, Germany). For protein reduction, 1 µL of 50 mM dithioerythritol was added and incubated for 30 min at 37°C. Cysteines were carbamidomethylated for 30 min by adding 2 µL of 0.1 M iodoacetamide and incubation for 30 min at RT. The first digestion step was performed with 100 ng Lys-C (FUJIFILM Wako Chemicals Europe GmbH, Germany) for 4 h. Then, samples were diluted with water to a final concentration of 1 M urea. A second digestion step using 200 ng modified porcine trypsin (Promega, WI, United States) was performed for 16 h at 37°C. For peptide identification and quantification, samples were injected on an UltiMate 3,000 nano LC system online coupled to a Q-Exactive HF-X instrument (Thermo Scientific). As liquid phases 0.1% formic acid in water (solvent A) and 0.1% formic acid in acetonitrile (solvent B) were used. Chromatographic separation was performed on an analytical column (PepMap RSLC C18, 75 μm × 50 cm, 2 µm particles, Thermo Scientific) at 250 nL/min with a 160-min gradient of 3%–25% of solvent B followed by 10-min increase to 40% and 5-min increase to 85%. MS spectra were acquired using a top 15 data-dependent acquisition method. MS and MS/MS were processed by Maxquant (v1.6.1.0) (Tyanova et al., 2016) using the Human subset of the UniProt database.

MaxQuant files were then loaded and analyzed in R using the package DEP (Zhang et al., 2018). We first filtered for proteins with a maximum of three missing values in one condition. Data was then normalized using VSN normalization. Imputation was conducted using quantile regression-based left-censored function (QRILC) in DEP package. The clustering of samples was done using PCA and cluster analysis. Proteins with a *p*-value <0.05 were considered differentially expressed. Data was also compared to the published dataset from Geyer et al. (2019). Functional and network analysis was performed using stringApp for Cytoscape.

### 2.5 Next-generation sequencing and transcriptomic analysis

We analyzed the platelet transcriptome in 9 AD patients, 9 Old and 9 Young controls. Total RNA was extracted by lysis with TRIzol Reagent (Thermo Fisher Scientific), separation with chloroform, and precipitation with isopropyl alcohol, according to the manufacturer’s instructions. RNA quality was assessed using Qubit. Library preparation was performed by GENEWIZ (Azenta Life Sciences, South Plainfield, NJ) with depletion of rRNA and globin mRNA and were subsequently sequenced using Illumina HiSeq 2,500 platform generating 150 bp paired end reads.

Sequencing yielded between 20 and 30 million sequence reads per sample. Adapters were removed using Trimmomatic [v.0.39 (Bolger et al., 2014)] and further confirmed using FastQC [v0.11.5 (Andrews, 2010)]. Reads were then mapped on GRCh38 transcriptome index (release 96) using Salmon pipeline (Patro et al., 2017). Transcript to gene mapping was conducted using tximport R package. The R package DESEQ2 (Love et al., 2014) was then used for differential expression analysis. Genes were considered significantly differentially transcribed with *p*-value <0.05. Enrichment analysis was conducted using ClusterProfiler package (Wu et al., 2021a). We also compared our analysis with the transcriptomic platelet data from Supernat et al. (2021) and checked for overlapping data.

## 3 Results

### 3.1 Platelet activation in Alzheimer’s disease and aging

**FIGURE 2:**
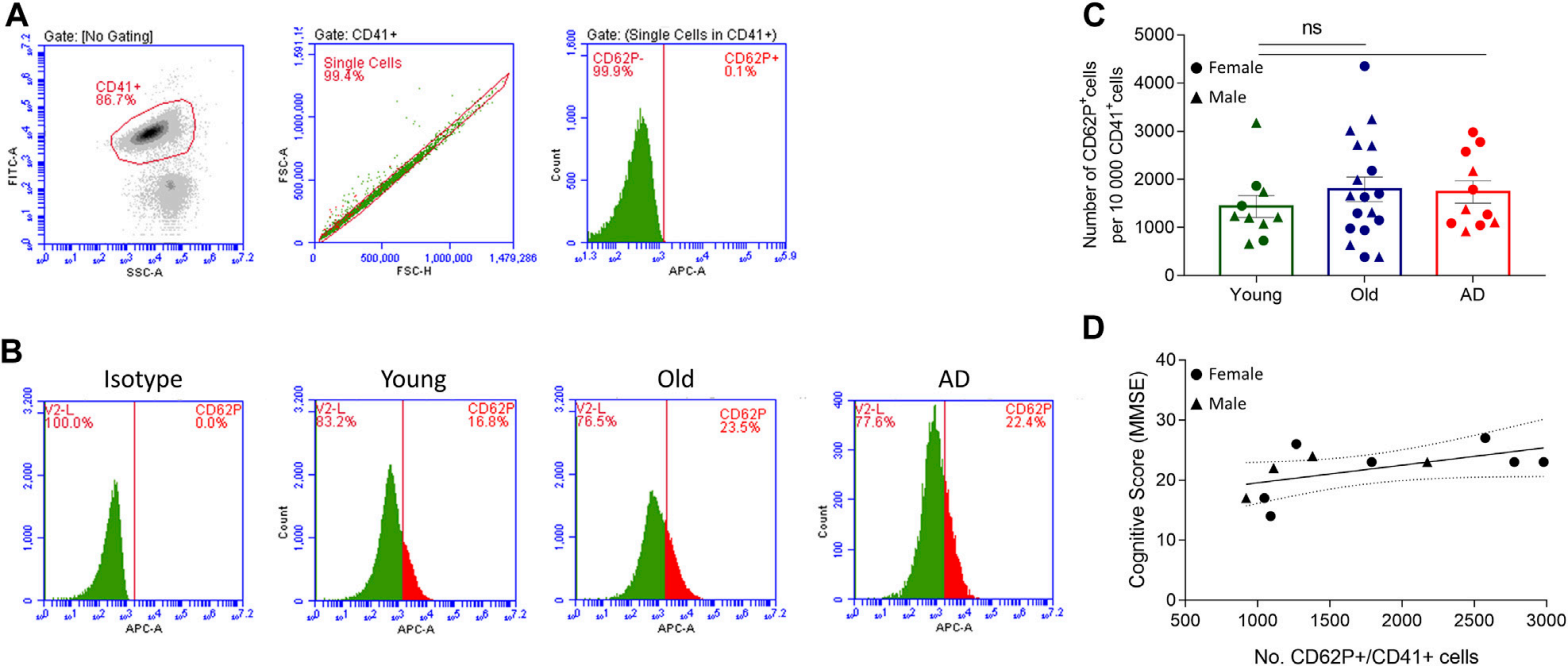
Platelet activation analysis. Two-color flow cytometry was used to detect the surface expression of CD62P, a platelet activation marker, in single CD41^+^ labelled platelets. **(A)** Gating strategy. **(B)** Representative flow-cytometry plots for IgG isotype and CD62P expression in control (young and old) and AD samples. **(C)** Platelet activation was similar between groups (*n* = 10 Young, 18 Old and 11 AD individuals). Data are shown as mean ± SEM, and statistical analysis was performed by one-way ANOVA with Holm-Šídák’s multiple comparisons test. **(D)** No correlation was found between platelet activation (number on CD62P^+^ cells/10 000 CD41^+^ cells) and MMSE score in AD individuals (*n* = 11; *r* ^2^ = 0.309; *p*-value = 0.0758, simple linear regression).

Alzheimer’s disease and aging are associated with alterations in platelet function (Sevush et al., 1998; Ciabattoni et al., 2007; Le Blanc and Lordkipanidzé, 2019). To assess platelet activation, we analyzed platelet surface expression of CD62P in platelets isolated from 11 AD patients, 18 non-demented elderly (Old) and 10 young healthy individuals (Young) using flow cytometry (Figures 2A–C). Overall, no statistically significant differences in platelet activation were observed between the three study groups (Figures 2B, C). However, AD and Old participants had a slightly higher (not statistically significant) basal platelet activation level than Young controls (Figures 2B, C). Furthermore, we found no statistically significant correlation between platelet activation levels and the MMSE score in AD patients (Figure 2D).

### 3.2 Proteomic profiling of AD and aged platelets indicates platelet activation

To analyze changes in the proteomic signature of AD and aged platelets, we used liquid chromatography coupled with high-resolution mass spectrometry (LC-MS/MS) to assess the proteome of blood isolated platelets from AD patients, Old and Young individuals.

**FIGURE 3:**
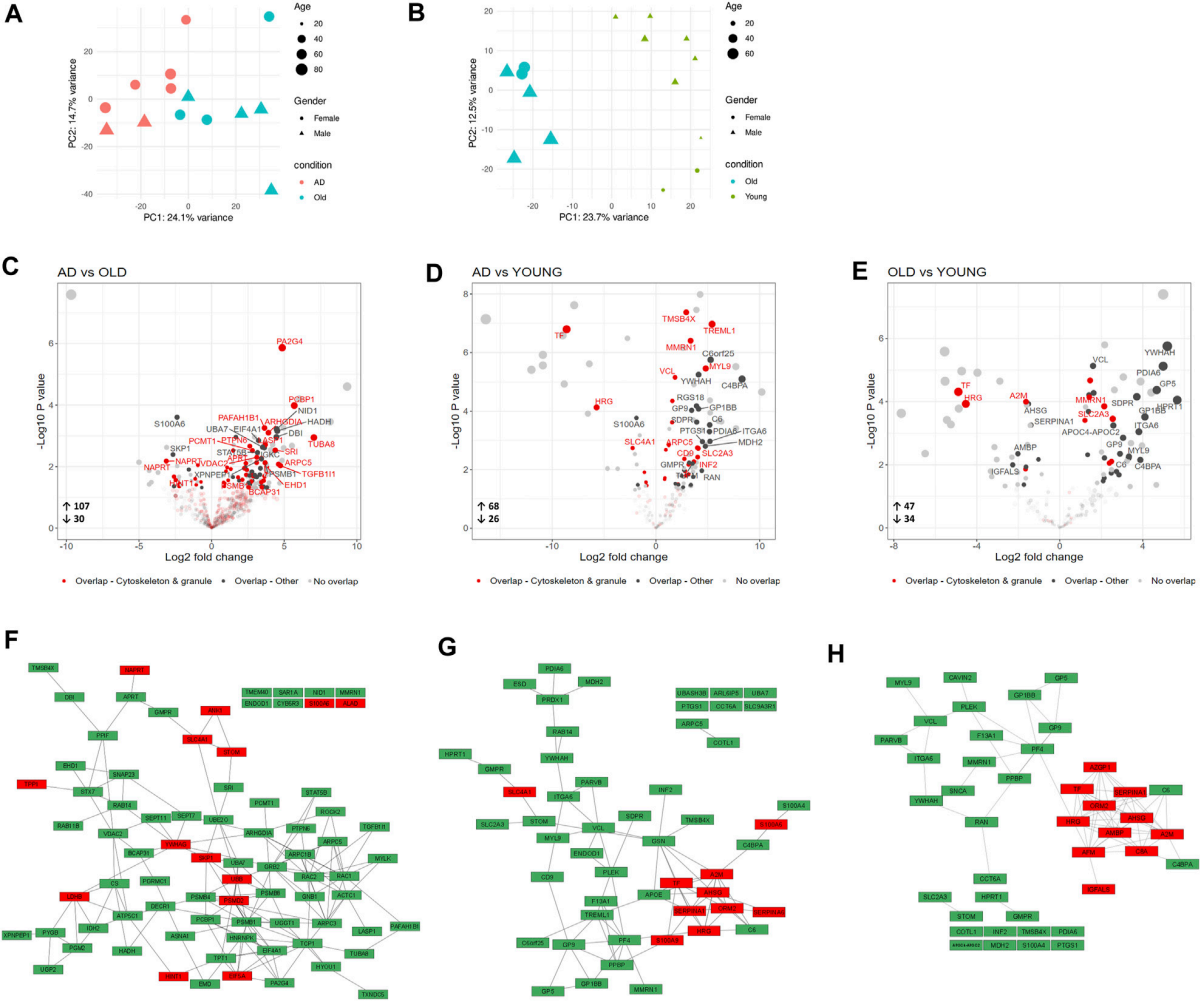
Proteomic analysis of blood isolated platelets in AD and aging. Principal component analysis of AD and non-demented elderly (Old) samples **(A)** and Old and Young samples **(B)**. Volcano plots showing differentially expressed proteins (DEPs, *p*-value < 0.05) in AD *versus* Old **(C)**, AD *versus* Young **(D)** and Old *versus* Young samples **(E)**. Platelet DEPs (proteins overlapping with the platelet proteome of Geyer et al. (2019)) related to the cytoskeleton and platelet granules are highlighted in red, and other platelet DEPs are highlighted in dark grey. Functional interactions among platelet DEPs in AD *versus* Old **(F)**, AD *versus* Young **(G)** and Old *versus* Young **(H)**. Upregulated platelet DEPs are displayed in green, and downregulated platelet DEPs in red. (*n* = 9 Young, 6-7 Old and 7 AD individuals).

Although the human platelet proteome contains 4,000–6,000 unique proteins (Burkhart et al., 2012; Lee et al., 2016; Geyer et al., 2019), we detected on average less than 500 proteins per sample. This unexpectedly low number of detected proteins may be related to the high amount of plasma proteins found in the samples. Although our proteomic analysis offers limited coverage of the AD and aged platelet proteomes, principal component analysis (PCA) clustered AD, Old and Young samples separately (Figures 3A, B). We also identified 137 differentially expressed proteins (DEPs, nominal *p*-value <0.05) in AD *versus* Old (Figure 3C), 94 DEPs in AD *versus* Young (Figure 3D), and 81 DEPs in Old *versus* Young samples (Figure 3E).

Since sample contamination with other blood cells is a major source of confounding effects in platelet omics studies (Wrzyszcz et al., 2017; Rowley et al., 2019; Chebbo et al., 2022), we compared DEPs between groups with the highly pure platelet proteome published by (Geyer et al., 2019) and limited functional analysis to the overlapping proteins.

Among the detected DEPs, 80 DEPs in AD *versus* Old (66 upregulated and 14 downregulated); 52 DEPs in AD *versus* Young (42 upregulated and 10 downregulated); and 42 DEPs in Old *versus* Young (31 upregulated and 11 downregulated) overlapped with Geyer’s platelet proteome (henceforth referred to as platelet DEPs) (Figures 3C–E—highlighted in dark grey and red). The complete lists of DEPs and platelet DEPs per comparison are presented in Supplementary Data S1.

To visualize the functional interaction of platelet DEPs, we used STRING for protein-protein interaction (PPI). Platelet DEPs in AD *versus* Old integrated a network of 79 nodes and 156 edges (nodes denote proteins and edges protein interactions, PPI enrichment: 1.0 × 10^−16^). Similarly, platelet DEPs formed a network of 52 nodes and 80 edges (PPI enrichment: <1.0 × 10^−16^) in AD *versus* Young samples; and a network of 42 nodes and 77 edges in Old *versus* Young samples (PPI enrichment: <1.0 × 10^−16^). Overall, platelet DEPs were functionally correlated (Figures 3F–H).

In AD *versus* Old samples, biological processes significantly represented included processes related to platelet function, such as actin cytoskeleton organization (ARPC3, RAC2, ACTC1, ARPC5, ROCK2, RAC1, TMSB4X, PAFAH1B1, ARPC1B) and exocytosis, but also several processes related with the immune system and neutrophil mediated immunity (Supplementary Data S1). Pathway analysis mapped several platelet DEPs to platelet activation, signaling and aggregation (ENDOD1, GNB1, GRB2, RAC1, RAC2, MMRN1, PTPN6, TMSB4X) (Supplementary Data S1), regulation of actin cytoskeleton (ARPC3, RAC2, ARPC5, ROCK2, MYLK, TMSB4X, ARPC1B) (Table 2). Proteasome and ubiquitin mediated proteolysis were also significantly enriched pathways in AD *versus* Old samples (UBB, UBA7, UBE2O, PSMB1, PSMB4, PSMB8, PSMD2, SKP1, VDAC2) (Table 2 and Supplementary Data S1). Several platelet DEPs were also mapped to pathways related to immune response, such as neutrophil degranulation (PYGB, PGRMC1, SNAP23, PSMB1, STOM, ARPC5, PA2G4, PSMD2, RAC1, CYB5R3, RAB14, APRT, TXNDC5, PGM2, ALAD, PTPN6, NAPRT) (Table 2).

**TABLE 2:** List of the top 5 significantly enriched Reactome and KEGG pathways and their corresponding proteins in AD *versus* Old platelets sorted after adjusted *p*-value.

*Reactome Pathway*	Proteins	FDR value
Innate Immune System	PYGB, PGRMC1, ARPC3, SKP1, RAC2, SNAP23, PSMB1, STOM, PSMB4, ARPC5, PA2G4, UBB, PSMD2, UBA7, RAC1, CYB5R3, RAB14, PSMB8, APRT, TXNDC5, PGM2, GRB2, ALAD, ARPC1B, PTPN6, NAPRT	4.72E-11
Immune System	PYGB, PGRMC1, ARPC3, SKP1, RAC2, SNAP23, PSMB1, STOM, PSMB4, STAT5B, EIF4A1, ARPC5, PA2G4, UBB, PSMD2, TCP1, UBE2O, UBA7, TUBA8, RAC1, CYB5R3, RAB14, PSMB8, APRT, TXNDC5, PGM2, GRB2, ALAD, ARPC1B, PTPN6, BCAP31, NAPRT	1.41E-09
Neutrophil degranulation	PYGB, PGRMC1, SNAP23, PSMB1, STOM, ARPC5, PA2G4, PSMD2, RAC1, CYB5R3, RAB14, APRT, TXNDC5, PGM2, ALAD, PTPN6, NAPRT	5.92E-09
Signaling by the B Cell Receptor (BCR)	SKP1, PSMB1, PSMB4, UBB, PSMD2, PSMB8, GRB2, PTPN6	1.24E-05
RHO GTPase Effectors	ARPC3, RAC2, ARPC5, YWHAG, ROCK2, TUBA8, RAC1, MYLK, GRB2, PAFAH1B1, ARPC1B	1.37E-05
*KEGG Pathway*		
Parkinson disease	PPIF, PSMB1, PSMB4, UBB, PSMD2, UBA7, TUBA8, ATP5C1, VDAC2	2.30E-04
Prion disease	PPIF, RAC2, PSMB1, PSMB4, PSMD2, TUBA8, ATP5C1, VDAC2	2.30E-03
Proteasome	PSMB1, PSMB4, PSMD2, PSMB8	2.90E-03
Regulation of actin cytoskeleton	ARPC3, RAC2, ARPC5, ROCK2, MYLK, TMSB4X, ARPC1B	2.90E-03
Shigellosis	ARPC3, SKP1, SEPT11, ARPC5, UBB, ROCK2, ARPC1B	2.90E-03

In AD *versus* Young samples, significantly enriched gene ontology (GO) terms included platelet alpha granule lumen (e.g., TMSB4X, PPBP, PF4, MMRN1), platelet activation (GP1BB, VCL, CD9, GP9, PF4, PLEK, GP5, MMRN1, MYL9, TREML1, UBASH3B), regulation of platelet activation (GP1BB, CD9, GP9, PLEK, GP5, MMRN1, UBASH3B) and actin cytoskeleton organization (COTL1, TMSB4X, SLC9A3R1, ARPC5, PLEK, INF2, MYL9, PARVB) (Supplementary Data S1). Similarly, enriched GO terms in Old *versus* Young samples included platelet alpha granule (TMSB4X, AHSG, PPBP, SERPINA1, F13A1, A2M, PF4, HRG, ORM2, MMRN1, SNCA), platelet activation (GP1BB, VCL, GP9, PF4, HRG, PLEK, GP5, MMRN1, MYL9), regulation of platelet activation (GP1BB, GP9, HRG, PLEK, GP5, MMRN1) (Supplementary Data S1). Pathway analysis also identified platelet activation, signaling and aggregation and platelet degranulation as significantly enriched pathways in AD *versus* Young (Table 3) and Old *versus* Young samples (Table 4).

**TABLE 3:** List of the top 5 significantly enriched Reactome and KEGG pathways and their corresponding proteins in AD *versus* Young platelets sorted after adjusted *p*-value.

*Reactome Pathway*	Proteins	FDR value
Platelet activation, signaling and aggregation	VCL, HRG, PLEK, F13A1, ENDOD1, PPBP, PF4, GP9, A2M, C6orf25, TMSB4X, CD9, MMRN1, GP1BB, GP5, TF, AHSG, ORM2, SERPINA1	4.74E-19
Platelet degranulation	VCL, HRG, PLEK, F13A1, ENDOD1, PPBP, PF4, A2M, TMSB4X, CD9, MMRN1, TF, AHSG, ORM2, SERPINA1	1.42E-17
Hemostasis	VCL, HRG, PLEK, F13A1, ENDOD1, PPBP, PF4, GP9, A2M, C6orf25, TMSB4X, CD9, MMRN1, GP1BB, GP5, TF, ITGA6, AHSG, ORM2, SERPINA1	2.27E-14
Formation of Fibrin Clot (Clotting Cascade)	F13A1, PF4, GP9, A2M, GP1BB, GP5	8.42E-07
Neutrophil degranulation	SLC2A3, VCL, COTL1, STOM, ARPC5, PPBP, S100A9, GSN, RAB14, AHSG, ORM2, SERPINA1	1.39E-06
*KEGG Pathway*		
Complement and coagulation cascades	C6, F13A1, A2M, C4BPA, SERPINA1	0.0011
Hematopoietic cell lineage	GP9, CD9, GP1BB, GP5, ITGA6	0.0011
Regulation of actin cytoskeleton	VCL, MYL9, ARPC5, GSN, TMSB4X, ITGA6	0.0024
ECM-receptor interaction	GP9, GP1BB, GP5, ITGA6	0.0087
Platelet activation	GP9, PTGS1, GP1BB, GP5	0.0233

**TABLE 4:** List of the top 5 significantly enriched Reactome and KEGG pathways and their corresponding proteins in Old *versus* Young platelets sorted after adjusted *p*-value.

*Reactome Pathway*	Proteins	FDR value
Platelet activation, signaling and aggregation	VCL, HRG, PLEK, F13A1, PPBP, PF4, GP9, A2M, TMSB4X, MMRN1, GP1BB, GP5, TF, AHSG, ORM2, SERPINA1	4.00E-16
Platelet degranulation	VCL, HRG, PLEK, F13A1, PPBP, PF4, A2M, TMSB4X, MMRN1, TF, AHSG, ORM2, SERPINA1	1.65E-15
Hemostasis	VCL, HRG, PLEK, F13A1, PPBP, PF4, GP9, A2M, TMSB4X, MMRN1, GP1BB, GP5, TF, ITGA6, AHSG, ORM2, SERPINA1	1.78E-12
Formation of Fibrin Clot (Clotting Cascade)	F13A1, PF4, GP9, A2M, GP1BB, GP5	2.21E-07
Intrinsic Pathway of Fibrin Clot Formation	GP9, A2M, GP1BB, GP5	1.10E-04
*KEGG Pathway*		
Complement and coagulation cascades	C6, F13A1, A2M, C8A, C4BPA, SERPINA1	1.06E-05
ECM-receptor interaction	GP9, GP1BB, GP5, ITGA6	0.0074
Hematopoietic cell lineage	GP9, GP1BB, GP5, ITGA6	0.0074
Platelet activation	GP9, PTGS1, GP1BB, GP5	0.0127

**FIGURE 4:**
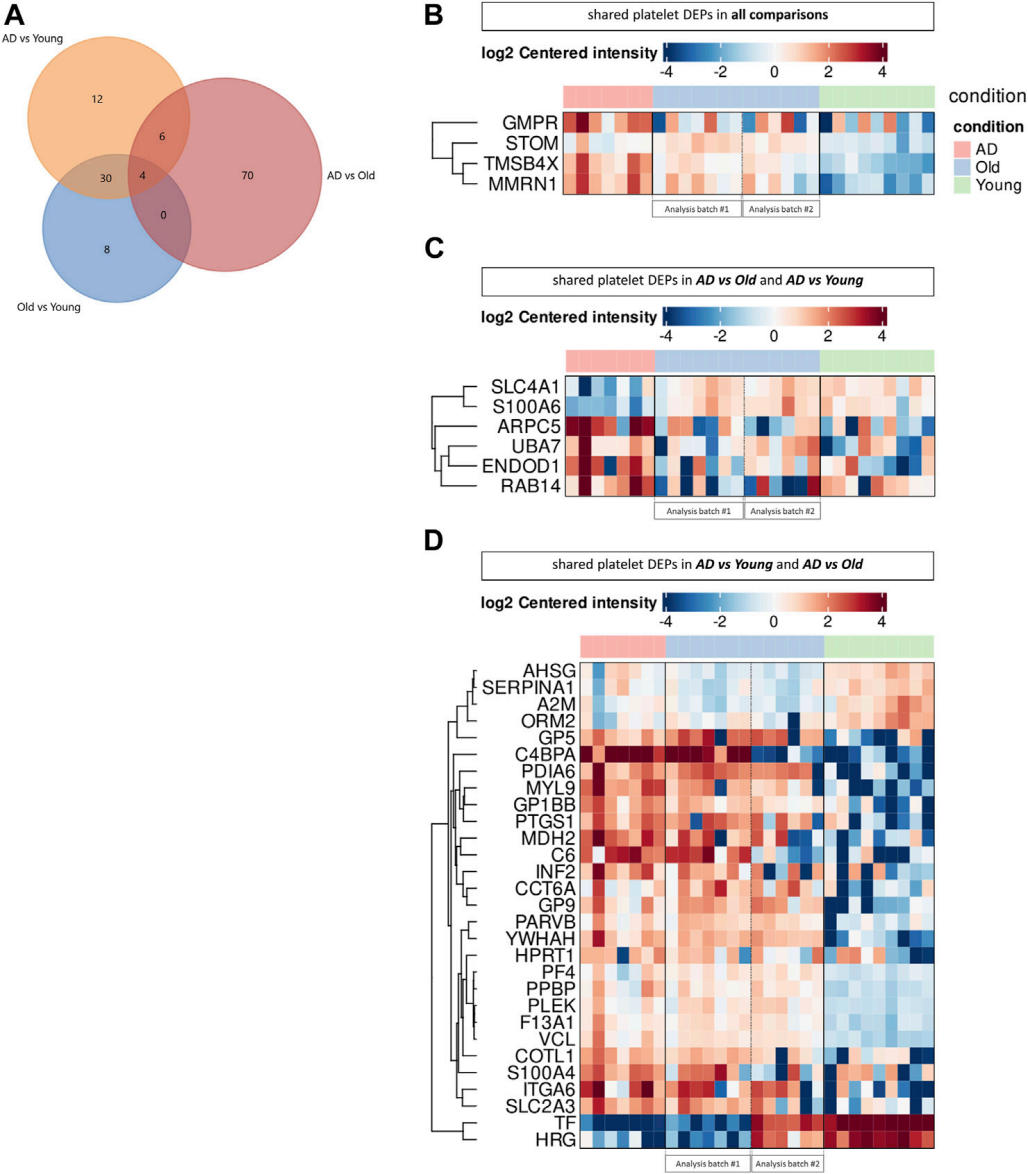
Comparison of the platelet proteome in AD and aging. **(A)** Venn diagram showing the number of platelet differential expressed proteins (DEPs) overlapping between comparisons. Heatmaps display the expression of shared platelet DEPs across all comparisons **(B)**, between AD *versus* Old and AD *versus* Young **(C)** and AD *versus* Young and AD *versus* Old **(D)**. (*n* = 9 Young, 6-7 Old and 7 AD individuals). Proteomic analysis was performed in two batches (batch #1: AD vs. Old; batch #2: Old vs. Young), using the same cohort of Old samples for differential protein expression analysis, as depicted in the heatmaps.

As AD and Old individuals showed the dysregulation of similar biological processes and pathways, we next investigated the presence of shared proteins between comparisons (Figure 4). Across all comparisons, we identified 4 shared platelet DEPs: STOM, MMRN1, TMSB4X, and GMPR (Figures 4A, B). Stomatin (STOM) was upregulated in AD *versus* Young and Old *versus* Young, but downregulated in AD *versus* Old, whereas the other three proteins were upregulated in AD (AD *versus* Old and AD *versus* Young) and aged samples (Old *versus* Young). Stomatin (STOM) is an abundant component of platelet alpha granule lipid rafts (Mairhofer et al., 2002), involved in membrane fusion during platelet granule exocytosis (Komatsuya et al., 2020). Multimerin 1 (MNRN1) is an alpha granule glycoprotein that supports the adhesion of activated platelets to the vessel wall (Tasneem et al., 2009). Thymosin β4 (TMSB4X) is a cytoplasm protein that binds G-actin and sequesters actin monomers (Xue et al., 2014), which can be rapidly mobilized for actin polymerization (Nachmias et al., 1993) and actin cytoskeleton reorganization upon platelet activation (Scheller et al., 2021). Lastly, guanosine monophosphate reductase (GMPR) is a negative regulator of GTP production (Wolff et al., 2022). GTP and GTP-binding proteins (i.e., heterotrimeric G proteins and small GTPases) are essential for signaling transduction, cytoskeletal remodulation and granule exocytosis upon platelet activation (Aslan and McCarty, 2013). Interestingly, we also observed the upregulation of hypoxanthine phosphoribosyltransferase 1 (HPTR1), a positive regulator of GDP and GTP synthesis (Wolff et al., 2022) among elderly (AD *versus* Young and Old *versus* Young); and GNB1, the beta subunit of G proteins, and of several GTPases (RAC1, RAC2, SAR1A, RAB14 and Rab11B) and GTPases associated proteins (ARHGDIA and ROCK2) in AD (AD *versus* Old).

Between AD *versus* Old and AD *versus* Young we identified 10 overlapping platelet DEPs (Figures 4A–C), including secretory vesicle proteins (ARPC5, STOM, TMSB4X, RAB14, MMRN1), proteins with cytoskeletal binding function (ARPC5, TMSB4X, RAB14, S100A6, SLC4A1) and proteins involved in platelet degranulation (TMSB4X, ENDOD1, MMRN1).

The highest number of shared platelet DEPs (34 DEPs) was found when comparing AD *versus* Young and Old *versus* Young (Figures 4A, B, D). These DEPs included several upregulated proteins involved in the adhesion of platelets to sites of vascular injury (GB1BB, GP5 and GP9, structural subunits of the GPIb-IX-V platelet receptor, and the integrin ITGA6, part of integrin receptor α6β1) and actin cytoskeleton dynamics (COTL1, INF2, MYL9, PARVB, and TMSB4X, VCL). Several platelet alpha granule proteins were also similarly dysregulated in AD and Old samples, including downregulation of A2M, AHSG, HRG, ORM2, SERPINA1 and upregulation of F13A1, MMRN1, PF4, PPBP, TMSB4X.

Summarizing our proteomics data, platelets in AD and aging showed dysregulation of several proteins linked to platelet activation and degranulation, including cytoskeleton and alpha-granule proteins. Furthermore, AD samples were enriched in platelet DEPs related to the ubiquitin-proteasome system.

### 3.3 Transcriptomic profiling of AD and aged platelets suggests dysregulation of proteolysis pathways

**FIGURE 5:**
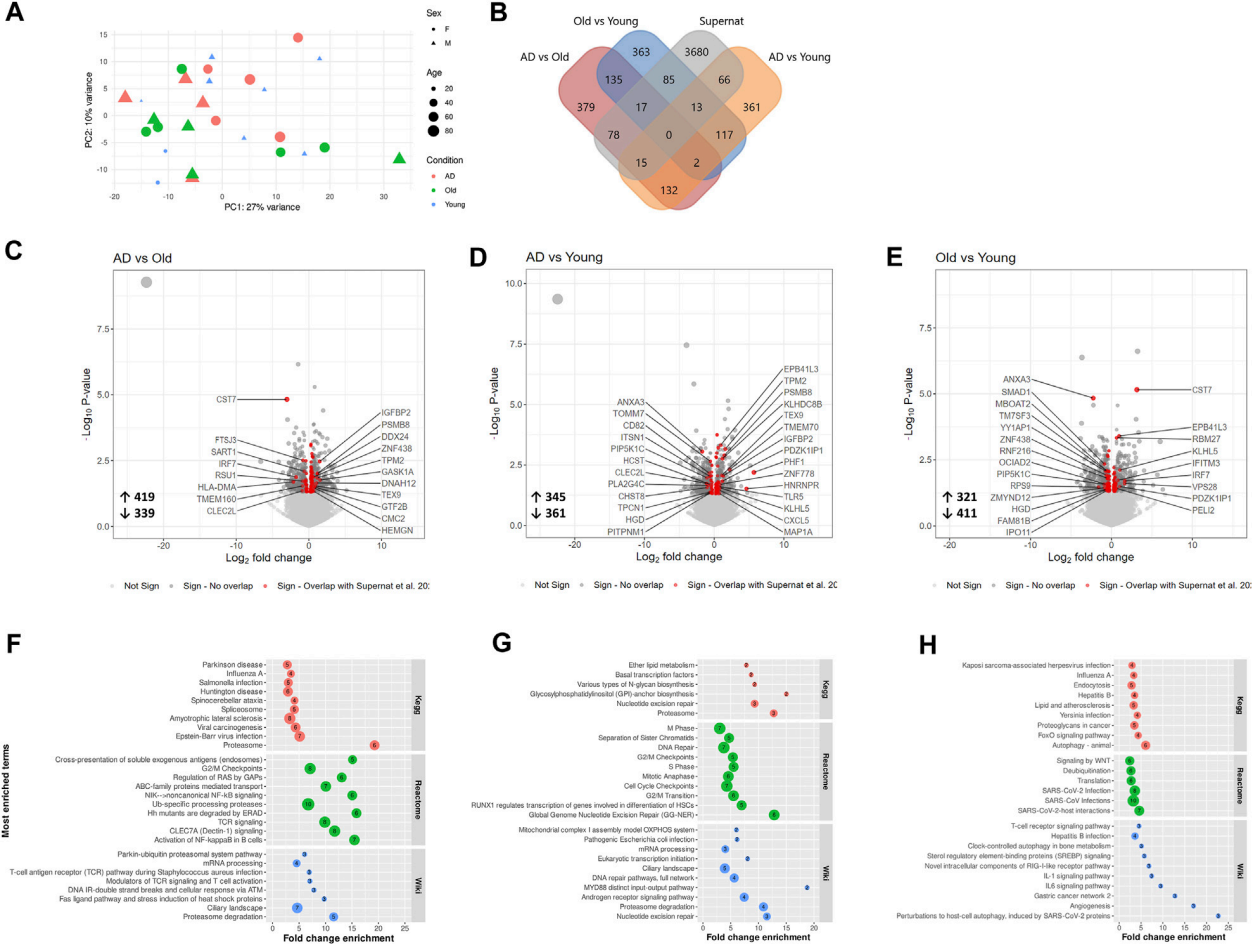
Transcriptomic analysis of blood isolated platelets in AD and aging. **(A)** Principal component analysis of AD, non-demented elderly (Old) and Young samples. **(B)** Overlap between differentially expressed transcripts (DETs, *p*-value < 0.05) detected across comparisons and the platelet transcriptomic dataset of Supernat et al. (2021). Volcano plots showing differentially expressed transcripts (DETs, *p*-value < 0.05) in AD *versus* Old **(C)**, AD *versus* Young **(D)** and Old *versus* Young samples **(E)**. Platelet DETs (messenger RNA transcripts overlapping with the platelet transcriptome of Supernat et al. (2021)) are highlighted in red, and the ones with log2 fold change >0.65 are labelled with their respective gene name. Pathway analysis of platelet DETs in AD *versus* Old **(F)**, AD *versus* Young **(G)** and Old *versus* Young **(H)**. (*n* = 9 individuals/group).

To investigate changes in the platelet transcriptome, we analyzed blood isolated platelets from AD, Old and Young individuals using RNA sequencing (Figure 5). We identified 758 differentially expressed messenger RNA transcripts (DETs, nominal *p*-value <0.05) in AD *versus* Old samples, 706 DETs in AD *versus* Young samples, and 732 DETs in Old *versus* Young samples (Supplementary Data S2). To verify the platelet specificity of these DETs, we compared DETs between groups with the platelet transcriptome dataset of 204 healthy donors reported by (Supernat et al., 2021) (Figure 5B). We identified 110 DETs in AD *versus* Old samples (80 upregulated and 30 downregulated); 94 DETs in AD *versus* Young samples (64 upregulated and 30 downregulated); and 115 DETs in Old *versus* Young samples (50 upregulated and 65 downregulated) overlapping with Supernat’s platelet transcriptome (henceforth referred as platelet DETs) (Figures 5C–E, colored in red). The complete lists of DETs and platelet DETs per comparison are presented in Supplementary Data S2.

To understand the biological significance of platelet DETs, we performed GO term enrichment and pathway analysis. In AD *versus* Old samples, platelet DETs were associated with cellular catabolic processes (Supplementary Data S2), and proteasome and proteasome degradation were the most significantly enriched KEGG and Wiki pathways (Figure 5F). Enriched platelet DETs associated with proteasome included PSME4, POMP, PSMD11, PSMB8, PSMD12 (upregulated) and PSMC2 (downregulated). Five of these proteasome-related DETs are present in all Reactome pathways depicted in Figure 5F. Interestingly, other platelet DETs related to ubiquitin dependent proteolysis or protein ubiquitination (UBA3, USP15, NUB1, HECTD1 and HERC2) were also upregulated in AD samples. In AD *versus* Young, platelet DETs were associated with nuclear processes such as mitotic cell cycle and nucleotide excision repair. However, the most significantly enriched Wiki pathway was proteasome degradation (upregulation: PSMB8, PSMD12; downregulation: PSMB7) (Supplementary Data S2 and Figure 5G).

In Old *versus* Young samples, functional analysis suggested a dysregulation of cellular and protein metabolic processes, with autophagy, regulation of autophagy and deubiquitination being among the most significantly enriched processes and pathways (Supplementary Data S2 and Figure 5H). Autophagy-related platelet DETs dysregulated in Old *versus* Young samples included ITPR1, MTDH, VPS13C, SUPT20H, MAP3K7, TRIM22 (upregulated in Old), and AKT1, EXOC7, STK11, LARP1, HGS, RB1CC1, VPS28 (downregulated in Old).

**FIGURE 6:**
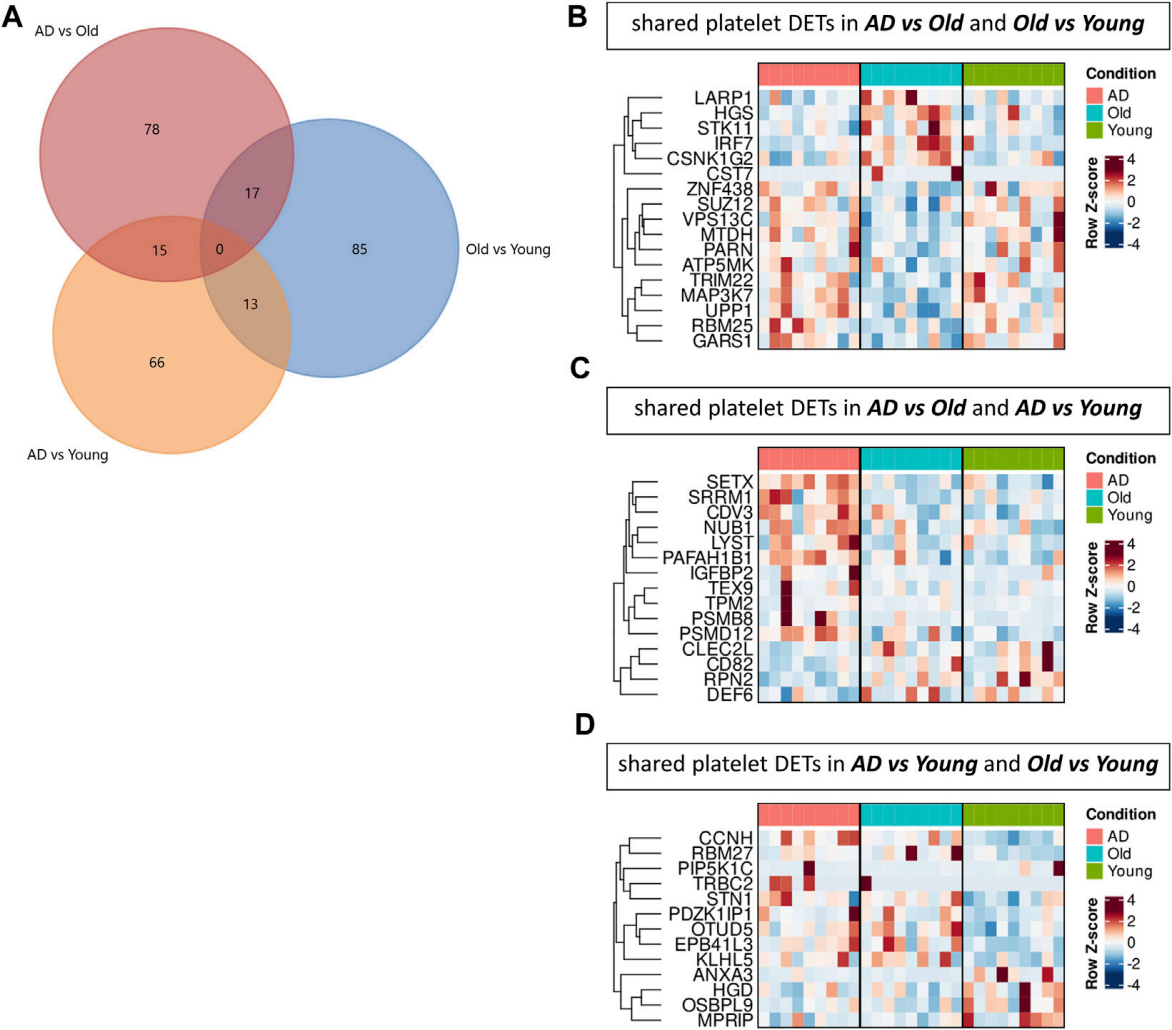
Comparison of platelet transcriptomic data sets in AD and aging. **(A)** Venn diagram showing the number of platelet differential expressed transcripts (DETs) overlapping between comparisons. Heatmaps display the expression of shared platelet DETs between AD *versus* Old and Old *versus* Young **(B)**, AD *versus* Old and AD *versus* Young **(C)** and AD *versus* Young and AD *versus* Old **(D)**. (*n* = 9 individuals/group).

To explore AD and aging-specific changes in the platelet transcriptome, we next investigated the overlap of platelet DETs between comparisons (Figure 6). In AD *versus* Old and Old *versus* Young, we identified 17 overlapping platelet DETs regulated in opposing directions (i.e., transcripts upregulated in AD were downregulated in aging, and *vice versa*) (Figures 6A, B). Gene ontology analysis mapped several of these transcripts to autophagy (LARP1, MAP3K7, MTDH, TRIM22, VPS13C, HGS, STK11).

In AD *versus* Old and AD *versus* Young, we identified 15 platelet DETs similarly regulated (Figures 6A, C), including transcripts related to the proteasome (PSMB8, PSMD12, NUB1), autophagy and vesicular trafficking (LYST (Holland et al., 2014), SETX (Richard et al., 2021; Richard and Rosonina, 2021)), cytoskeleton (TPM2, PAFAH1B1 (Markus et al., 2020) and GTPase regulation (DEF6 (Oka et al., 2007)).

In AD *versus* Young and Old *versus* Young, we identified 13 platelet DETs similarly regulated (Figures 6A, D), including transcripts associated with the actin cytoskeleton (MPRIP) and integrin-cytoskeleton interaction (PIP5K1C (Wang et al., 2013)).

Summarizing our transcriptomic data, platelets in AD and aging showed dysregulation of several transcripts related to cellular catabolism. Samples from AD patients were enriched in DETs related to the ubiquitin-proteasome system, and samples from healthy elderly individuals were enriched in DETs related to autophagy.

### 3.4 Comparison of the transcriptomic and proteomic profiles of AD platelets

**FIGURE 7:**
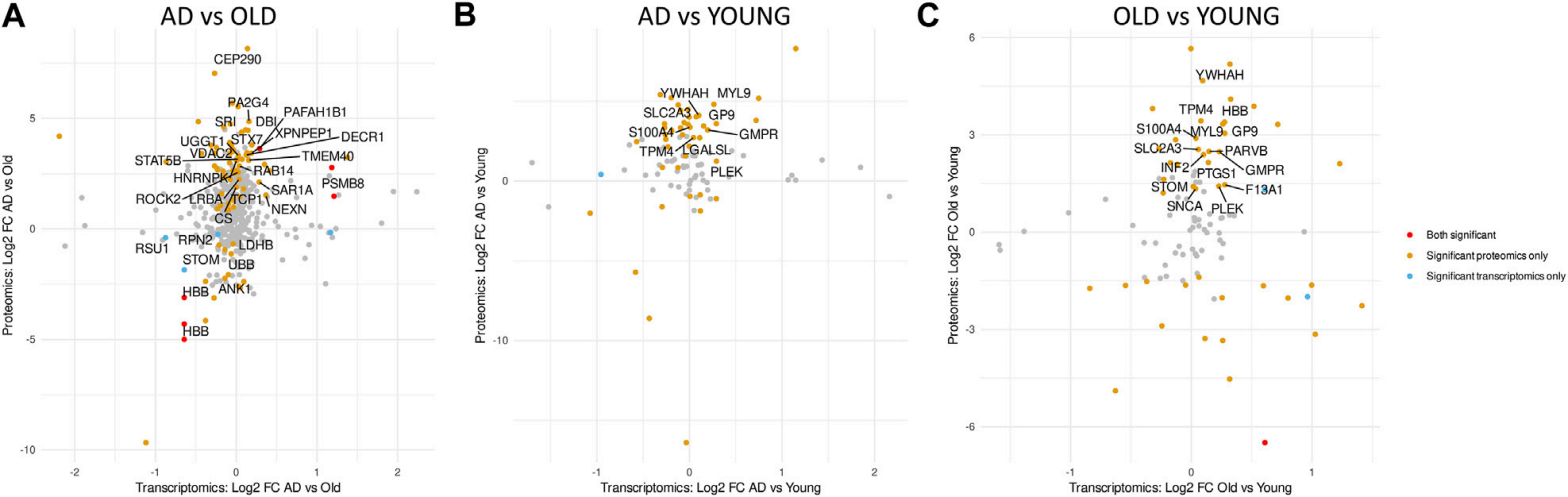
Comparative analysis of the transcriptome and proteome of AD platelets. Scatter plots showing overlapping differential expressed transcripts (DETs) and proteins (DEPs) in AD *versus* Old **(A)**, AD *versus* Young **(B)** and Old *versus* Young **(C)**. Red dots indicate overlapping DETs and DEPs with *p*-value < 0.05 in both omics datasets. Orange and blue dots indicate overlapping DETs and DEPs that are only significantly dysregulated at the protein or transcript level, respectively. Overlapping DETs and DETs with *p*-value > 0.05 in both datasets are represented in grey. Only platelet DETs are labelled with the corresponding gene name.

The abundance of mRNA transcripts and their coding proteins correlates weakly in platelets. However, for most proteins, platelets also express the corresponding mRNA transcript (Macaulay et al., 2005; Rowley et al., 2019). To assess the concordance between mRNA and protein expression changes, we compared log2 fold changes of all identified DETs and DEPs per comparison (regardless of *p*-value) (Figure 7). In AD *versus* Old samples, we identified 443 overlapping DET/DEPs, from which 4 were significantly differentially expressed only at the transcript level (Figures 7A—blue dots), 76 were significantly differentially expressed only at the protein level (Figures 7A—orange dots), and 6 were significantly differentially expressed at transcript and protein levels (Figures 7A—red dots). However, only two of the DET/DEPs significantly differently expressed at the transcript and protein level were found in Supernat and Geyer’s datasets: PSMB8 (proteasome 20S subunit beta 8) and PAFAH1B1 (platelet activating factor acetylhydrolase 1B subunit), both significantly upregulated at the transcript and protein level in AD samples. In AD *versus* Young (Figure 7B) and Old *versus* Young samples (Figure 7C), we observed little overlap between DETs and DEPs, and none of the overlapping DET/DEPs significantly differently expressed at the transcript and protein level were present in Supernat and Geyer’s datasets.

Previous proteomic studies have identified the dysregulation of several cytoskeletal proteins in AD platelets (Veitinger et al., 2014; González-Sánchez et al., 2018; Reumiller et al., 2018). Since PAFAH1b1 regulates dynein, a microtubule-based motor protein (Markus et al., 2020) involved in platelet shape change upon activation (Cuenca-Zamora et al., 2019), its upregulation at protein and transcript level makes it an interesting candidate for validation. To confirm the expression of PAFAH1B1 by platelets, we used immunocytochemistry to assess its expression in wild-type (WT) and APP Swedish PS1 dE9 (APP-PS1) mouse platelets (Supplementary Figure S1). APP-PS1 mice are genetically engineered to overproduce amyloid beta and replicate several features of AD, including amyloid plaque pathology, gliosis and cognitive deficits (Jankowsky et al., 2004; Lalonde et al., 2005; Janota et al., 2015; Unger et al., 2016; Unger et al. 2018a; Unger et al. 2018b; Unger et al. 2020). In this model, platelets produce human Abeta1-40 and Abeta1-42 (Wu et al., 2021b; Sun et al., 2021), present signs of platelet activation and similar ultrastructural abnormalities to those of platelets from patients of different neurodegenerative conditions (Kniewallner et al., 2020). We observed that mouse platelets express PAFAH1B1 [and PSMB8 (Supplementary Figure S1A)], but its expression was similar in platelets isolated from 18 months-old APP-PS1 mice, age-matched and young (4 months old) WT mice (Supplementary Figures S1B, C).

## 4 Discussion

Alzheimer’s disease and aging are associated with a hyperactive platelet phenotype (Sevush et al., 1998; Davizon-Castillo et al., 2019; Wiest et al., 2019) (also reviewed in (Le Blanc and Lordkipanidzé, 2019; Faria et al., 2020; Ferrer-Raventós and Beyer, 2021). However, the biological mechanisms supporting these phenotypic changes are poorly understood. To investigate whether changes in platelet reactivity in AD and aging result from an altered platelet molecular signature, we analyzed the activation, proteome, and transcriptome of platelets in AD patients, non-demented elderly, and young individuals. AD and aged individuals showed similar platelet activation profiles based on the expression of the activation marker CD62P but had a proteomic signature suggestive of increased platelet activation compared with young controls. Our transcriptomic analysis pointed out the dysregulation of transcripts associated with proteasomal degradation in AD and autophagy in aging, two essential processes for platelet function, but the implications of its dysregulation at the transcriptomic level remain unclear.

In our study cohort, AD and non-demented aged individuals showed a tendency towards higher levels of CD62P^+^ activated platelets (*p*-value > 0.05) and upregulation of several proteins linked to platelet activation and hemostasis in comparison with young subjects, including structural subunits of adhesive receptors (GP1BB, GP5, GP9, ITGA6); actin binding proteins involved in cytoskeletal remodeling (TMSB4X, VCL, PARVB, MYL9, COTL1, INF2); and granule proteins involved in coagulation (F13A1, PF4), platelet adhesion and aggregation (MMRN1, PDIA6, COTL1). AD and aged individuals also showed downregulation of proteins involved in the negative regulation of blood coagulation (A2M, HRG, TF). These changes suggest dysregulated platelet activation in aging and AD and strengthen the evidence of aging-associated platelet hyperactivity and elevated thrombotic risk.

The seminal work of (Sevush et al., 1998) identifying platelet hyperactivity in AD reported elevated platelet surface expression of CD62P and higher levels of circulating platelet and platelet-leukocyte aggregates in AD patients compared with healthy elderly individuals. However, like us, several subsequent studies have reported similar levels of CD62P platelet surface expression or percentages of circulating CD62P^+^ platelets in AD and age-matched control individuals (Kozubski et al., 2002; Stellos et al., 2010; Järemo et al., 2013; Plagg et al., 2015). Platelet function varies across individuals, and cohorts of healthy individuals can be stratified based on their platelet function (Dunster et al., 2021). This high interindividual variability in platelet function might have prevented us and others from detecting differences in platelet function in small study cohorts. The study from Sevush et al. (1998)—the only one yet to show significant differences in CD62P expression between AD and age-matched individuals–is the largest study so far conducted to assess platelet function in AD. Interestingly, the results presented by Sevush et al., 1998 clearly demonstrated high interindividual variability in platelet surface expression of CD62P in the AD and control groups (Sevush et al., 1998).

Platelet activation is a complex process, and relying on CD62P alone as a phenotypic marker of platelet activation provides insight into only one of the events occurring during platelet activation: granule secretion. Previously, Davizon-Castillo et al. (2019) demonstrated that platelets from elderly individuals express higher levels of activated fibrinogen receptor GPIIb/IIIa than platelets from young individuals. However, like us, an earlier study (Campbell et al., 2018) reported a similar percentage of circulating CD62P+ platelets in young and elderly individuals. Additional studies are needed to understand better what aspects of platelet activation change in AD and aging. However, to achieve meaningful results, future studies must consider the recruitment of larger study cohorts and use more comprehensive approaches for assessing platelet function.

Although AD and aging have been associated with platelet hyperactivity, a growing body of evidence highlights that AD and aged platelets respond differently to agonists (Rao et al., 1996; Kozubski et al., 2002; Yu and Jia, 2009, Järemo et al. 2013; Wiest et al., 2019) and significantly differ in their proteomic (Zellner et al., 2012; Donovan et al., 2013; Veitinger et al., 2014; González-Sánchez et al., 2018; Reumiller et al., 2018; Yu et al., 2021), metabolomic (Oberacher et al., 2017) and transcriptomic profiles (Gámez-Valero et al., 2021). In this study, AD individuals showed upregulation of proteins linked to cytoskeleton reorganization (ARPC1B, ARPC3, ARPC5, MYLK, PAFAH1B1, RAC1, RAC2, ROCK2, TUBA8, TMBS4X), platelet adhesion (MMRN1), and cell signaling (GNB1, RAC1, RAC2, SAR1A, RAB14, Rab11B, ARHGDIA, ROCK2), changes that may contribute to platelet dysfunction in AD.

Platelet function strongly relies on a dynamic cytoskeleton, as platelet shape change is involved in granule release, platelet adhesion, and aggregation. In AD, previous platelet proteomic studies showed dysregulation of several actin cytoskeleton binding proteins, including increased levels of tropomyosin (Veitinger et al., 2014; Reumiller et al., 2018) and talin (González-Sánchez et al., 2018) and decreased levels of vinculin and moesin (González-Sánchez et al., 2018). Here, we identify the upregulation of PAFAH1B1 at protein and transcript levels in AD individuals. Platelet activating factor acetylhydrolase 1B subunit 1 (PAFAH1B1) is a key regulator of dyneins (Markus et al., 2020), microtubule motor proteins involved in cytoskeleton remodulation during platelet activation (Cuenca-Zamora et al., 2019; Markus et al., 2020). Increased levels of PAFAH1B1 might lead to altered cytoskeletal dynamics, impacting platelet function in AD. Interestingly, a previous platelet proteomic study (Yu et al., 2021) has also detected upregulation of dynactin subunit 1 (DCTN1), a protein interacting with dynein and PAFAH1B1 to promote microtubule motility, in AD platelets (Yu et al., 2021).

Although several studies have investigated changes in the proteomic profile of AD platelets (Zellner et al., 2012; Donovan et al., 2013; Veitinger et al., 2014; González-Sánchez et al., 2018; Reumiller et al., 2018; Yu et al., 2021), the transcriptomic profile of AD platelets remains largely unexplored. Platelets have a dynamic transcriptome, and transcriptomic alterations occur in aging and several diseases with functional implications (Davizon-Castillo et al., 2020). We observed the dysregulation of several transcripts related to the ubiquitin-proteasomal system in AD and autophagy in aged platelets, two proteolytic pathways involved in the regulation of platelet function (Feng et al., 2014; Gupta et al., 2014; Ouseph et al., 2015; Colberg et al., 2021).

Platelets possess an active proteasome system (Klockenbusch et al., 2014), and its inhibition impairs platelet aggregation (Colberg et al., 2021). Besides regulating protein content, the ubiquitin-proteasome system in platelets is involved in cell signaling (Unsworth et al., 2019) and cytoskeleton remodeling following platelet activation (Gupta et al., 2014). In AD individuals, we observed the dysregulation of several mRNA transcripts (AD *versus* Old: PSME4, POMP, PSMB8, PSMD11, PSMD12, PSMC2; AD *versus* Young: PSMB8, PSMD12 AND PSMB7) and proteins (AD *versus* Old: UBB, UBA7, UBE2O, PSMB1, PSMB4, PSMB8, PSMD2, SKP1) related with ubiquitin-proteasome system. Previous platelet proteomic studies have also demonstrated dysregulation of proteosome constituents [i.e., downregulation of PSMD6, PSMC6, PSMB1, PSMB2 (Donovan et al., 2013)] and ubiquitin mediated proteolysis in AD platelets (Yu et al., 2021). However, whether these transcriptomic and proteomic changes impact platelet proteasomal activity or contribute to platelet dysfunction in AD remains unknown.

We also observed the upregulation of the immunoproteasome catalytic subunit PSMB8 at transcript and protein levels in AD individuals. The immunoproteasome is an alternative proteasome form involved in major histocompatibility complex (MCH) class I antigen presentation and clearance of oxidant-damaged proteins (Johnston-Carey et al., 2015). Platelets have a constitutively active immunoproteasome, but its function is still unclear (Klockenbusch et al., 2014). Inflammation and oxidative stress induce the expression of the immunoproteasome (Johnston-Carey et al., 2015), and in the AD brain, the immunoproteasome, including the subunit PSMB8, is upregulated (Mishto et al., 2006; Orre et al., 2013). Platelets mirror several molecular alterations happening in the AD brain (Yu et al., 2022), which might also be the case with PSMB8 dysregulation. The functional and pathological implications of elevated PSMB8 levels in platelets require further investigation.

Autophagy is constitutively active in platelets and induced upon platelet activation, being essential for platelet aggregation (Feng et al., 2014; Ouseph et al., 2015). Several transcripts related to autophagy were upregulated (ITPR1, MTDH, VPS13C, SUPT20H, MAP3K7, TRIM22) and downregulated (AKT1, EXOC7, STK11, LARP1, HGS, RB1CC1, VPS28) in aged individuals in comparison with young controls, suggesting the dysregulation of autophagy in aged platelets. Interestingly, LARP1, MAP3K7, MTDH, TRIM22, VPS13C, HGS, and STK11 were regulated in opposing directions in Old *versus* Young and AD *versus* Young samples. Autophagy (and mitophagy - selective autophagy of damaged and unwanted mitochondria) is defective in aging and AD (Aman et al., 2021; Tran and Reddy, 2021). However, as we observed no changes in autophagy-related proteins in aged platelets, further studies are needed to investigate the functional implications of these alterations.

### 4.1 Limitations of the study

The contamination of platelet isolates by other blood cells is a major concern when performing platelet omics studies. In this study, we used a classical approach based on differential centrifugation for platelet isolation. Although we collected only the top part from the PRP fractions to avoid erythrocyte and leukocyte contamination, this approach still allows cell contaminants (Chebbo et al., 2022). To minimize the interference of potential contaminants, we focus our analysis on proteins and transcripts previously reported in reference platelet datasets. However, many identified platelet DEPs and DETs are also present in leukocytes, erythrocytes, and plasma, which could explain the enrichment of proteins associated with neutrophil-mediated immunity in AD *versus* Old samples. Although these neutrophil-associated proteins are expressed in platelets and might derive exclusively from platelets, we cannot exclude the possibility that they might have derived from contaminating neutrophils. Alternatively, the *ex vivo* formation of neutrophil extracellular traps (NETs) before platelet isolation could also justify the presence of these proteins (Tassi Yunga et al., 2022).

The lack of independent validation of the many identified and discussed platelet DEPs and DETs is also an important caveat of this study. Since this was a small exploratory study, we decided, like others (Pascovici et al., 2016; St John et al., 2021), to not correct for multiple comparisons. We used this approach to gain a broader overview of the molecular changes occurring in AD and aged platelets. However, this might have led us to regard false positives as true DEPs and DETs. For example, we detected the upregulation of PAFAH1B1 in AD samples at transcript and protein levels but failed to validate its upregulation in a widely used AD pre-clinical model. However, given the paramount urgency in better understanding AD pathology, the dysregulated proteins and transcripts identified in the blood of AD and elderly participants might provide new study material to understand dysregulated processes in AD and aging.

## 5 Conclusion

Our data strengthen the evidence that platelet activation is increased in aging and provide a first glimpse of platelet transcriptomic changes occurring in AD. Further studies are needed to explore the possible role of these alterations in platelet dysfunction in AD and their impact on disease progression.

## Data Availability

The data presented in the study are deposited on Zenodo and available through the link https://zenodo.org/record/7955730 or included in the Supplementary Table S3. The raw data supporting the conclusions of this article will be made available by the corresponding authors, without undue reservation.

## Supplementary material

Supplementary Figure S1 Mouse platelets express PAFAH1B1 and PSMB8. Mouse platelets in blood smears **(A)** and in platelet rich plasma **(B)** were immunostained for qualitative analysis of PAFAH1B1 (red) and PSMB8 (white) expression. CD41 (green) was used to visualize platelets, and DAPI (blue) was used as nucleus staining. Mouse platelets express PAFAH1B1 and PSMB8. Scale bars: 10 µm. **(C)** Platelets in platelet rich plasma of WT and APP-PS1 mice expressed similar levels of PAFAH1B1. Data are shown as mean ± SEM (n = 4/group, all males). Statistical analysis was performed by one-way ANOVA with Dunn’s multiple comparisons test.

Supplementary Table S1 Spreadsheet containing the lists of differentially expressed proteins per comparison, overlapping proteins with Geyer et al platelet proteomics dataset, and significantly enriched GO terms and pathways.

Supplementary Table S2 Spreadsheet containing the lists of differentially expressed transcripts per comparison, overlapping transcripts with Supernat et al platelet transcriptomics dataset, and significantly enriched GO terms and pathways.

Supplementary Table S3 Spreadsheet containing normalized DESeq2 counts from platelet RNAseq data.
