# Supplementary figures and images for: The platelet transcriptome and proteome in Alzheimer’s disease and aging: an exploratory cross-sectional study
Source: Front Mol Biosci. 2023 Jun 30;10:1196083. doi: 10.3389/fmolb.2023.1196083 (PMC10348715; doi:10.3389/fmolb.2023.1196083)

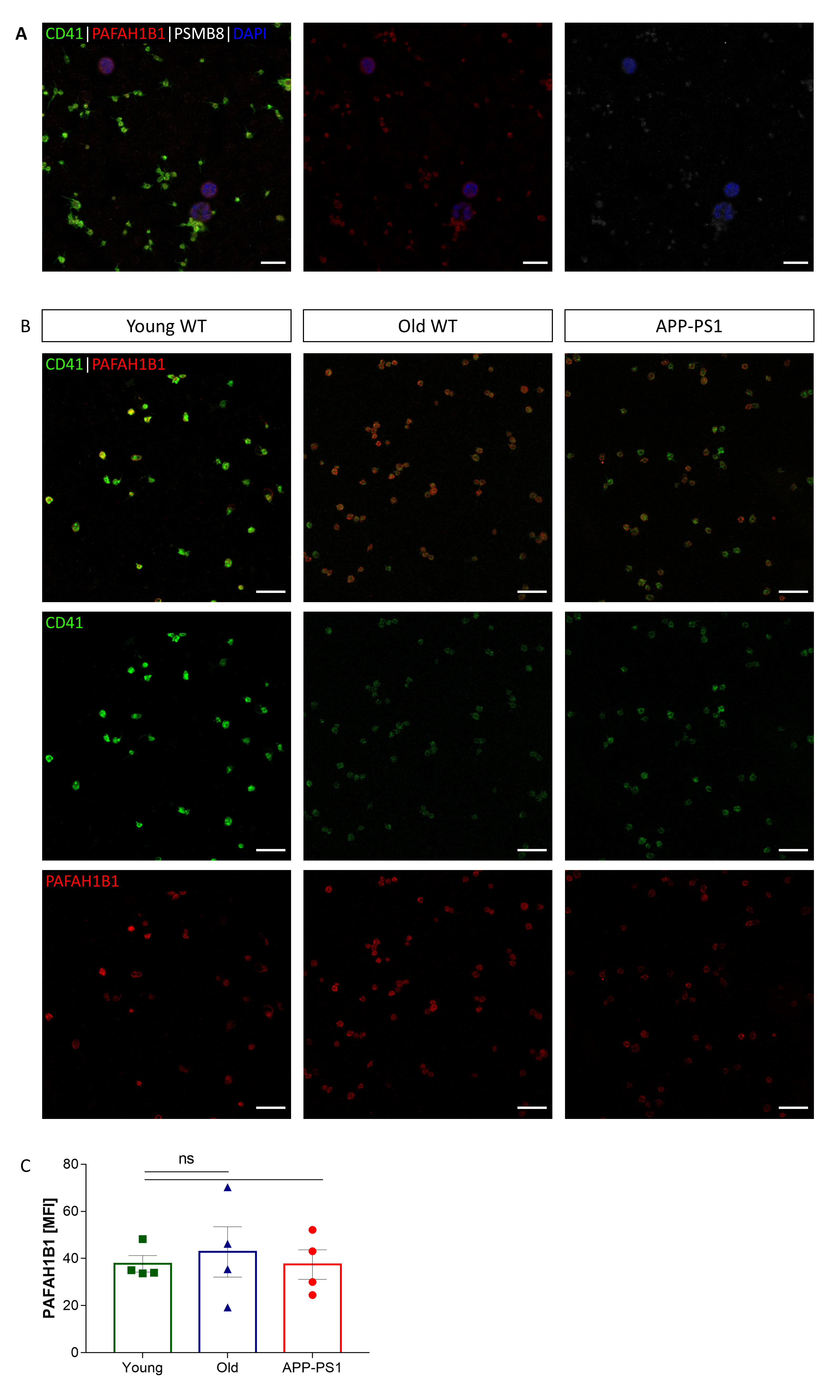

Supplement: Supplementary file 3 [file Image1.TIF]
