# Supplementary material for: The platelet transcriptome and proteome in Alzheimer’s disease and aging: an exploratory cross-sectional study
Source: Front Mol Biosci. 2023 Jun 30;10:1196083. doi: 10.3389/fmolb.2023.1196083 (PMC10348715; doi:10.3389/fmolb.2023.1196083)
Supplement: Supplementary file 5 [file DataSheet1.docx]

Supplementary Material

The platelet transcriptome and proteome in Alzheimer’s disease and aging: an exploratory cross-sectional study

Diana M Bessa de Sousa, Rodolphe Poupardin, Adam Schroer, Saul Villeda, Thomas Fröhlich, Vanessa Frey, Wolfgang Staffen, Heike Mrowetz, Barbara Altendorfer, Michael S Unger, Bernhard Iglseder, Bernhard Paulweber, Eugen Trinka, Janne Cadamuro, Martin Drerup, Katharina Schallmoser, Ludwig Aigner^*^, Kathrin M Kniewallner

*** Correspondence:** Kathrin M. Kniewallner, kathrin.drerup@pmu.ac.at

# Supplementary Material and Methods

## Expression of PAFAH1B1 and PSMB8 in mouse platelets

### Animals

We assessed the expression of PAFAH1B1 and PSMB8 in platelets isolated from 18 months old APP Swedish PS1 dE9 (APP-PS1) mice, age-matched (18-19 months old) and young (4 months old) wild-type mice (WT) (n=4/group, all male). APP-PS1 mice express a chimeric mouse/human mutant amyloid precursor protein (Mo/HuAPP695swe) and a mutant presenilin 1 (PS1-dE9) under the control of the mouse prion protein promoter (Mutant Mouse Resource and Research Center (MMRRC) strain #034832-JAX, The Jackson Laboratory) (Laboratory , Jankowsky, Slunt et al. 2001). At 18 months of age, APP-PS1 mice present severe amyloid plaque pathology, gliosis, cerebrovascular alterations, and cognitive deficits (Jankowsky, Fadale et al. 2004, Lalonde, Kim et al. 2005, Janota, Brites et al. 2015, Unger, Marschallinger et al. 2016, Unger, Marschallinger et al. 2018, Unger, Schernthaner et al. 2018, Unger, Li et al. 2020). Mice were housed in groups, in individually ventilated cages (IVC), under standard conditions at the Paracelsus Medical University with a constant 12-hour light/dark cycle, at 22 °C, RT and with *ad libitum* access to standard rodent chow (sniff® rodent maintenance chow 10mm pellets (#V1534)) and water. Animal breeding, handling, genotyping, and experiments were approved by local authorities (BMWFW-66.019/0011-WF/V/3b/2016 and 2020-0.078.469).

### Sample preparation and immunocytochemistry

Mice were anesthetized with a solution of ketamine (20.5 mg/mL; Ketamidor; Richter Pharma), xylazine (5.36 mg/mL; Chanazine; Chanelle), and acepromazine (0.27 mg/mL; Vanastress 10 mg/mL; Vana GmbH) in 0.9% sodium chloride. Following thoracotomy, blood was collected by cardiac puncture using ethylenediaminetetraacetic acid (EDTA; 0.1 M; Promega) coated syringes and immediately diluted 1:10 in EDTA. About 50µl of anticoagulated blood was used to prepare blood smears and the remaining volume to produce platelet-rich plasma (PRP). For that, whole blood was diluted in twice its volume with modified Tyrode’s buffer and centrifuged at 300 x g, for 5 min, RT. The complete PRP fraction and the top layer of the erythrocyte fraction were collected and centrifuged at 200 x g, for 12 min, RT. The top 2/3 of the PRP layer was then collected and applied onto slides for immunocytochemistry. Blood smears and PRP slides were fixed with 4% paraformaldehyde (PFA) for 15min at RT, washed 3x with 1x PBS and dried before storing at 4°C.

For immunocytochemistry, slides were washed with PBS, blocked with fish skin gelatin buffer (FSGB) containing 0,1% Triton (1h, RT) and incubated with primary antibodies (overnight, RT). The following primary antibodies were used: rat anti-CD41 (Abcam, ab33661, 1:300), goat anti-Lis1 (Abcam, ab117451, 1:150), rabbit anti-PSMB8 (Invitrogen, PA5-22290; 1:100). After extensive washing in PBS, slides were incubated with secondary antibodies (3h, RT). Following antibodies were used (all 1:1000): donkey anti-rat Alexa Fluor 488 (Invitrogen, A21208), donkey anti-goat Alexa Fluor 568 (Invitrogen, A11057), donkey anti-rabbit Alexa Fluor 647 (Invitrogen, A31537). Nucleus counterstaining was performed with 4′,6′-diamidino-2-phenylindole dihydrochloride hydrate (DAPI; 1 mg/mL; 1:2000; Sigma-Aldrich).

### Microscopy and image analysis

Confocal laser scanning microscopy was performed using a LSM 710 from Zeiss for qualitative and quantitative microscopic analysis of platelets. Images were taken at 63× and 100× magnification.

Quantitative analysis of platelet PAFAH1B1 expression was performed using Fiji/ImageJ (version 2.9.0/1.53t) based on the protocol published by (Shihan, Novo et al. 2021). To select CD41^+^ regions of interest (ROIs), we first used the “Threshold” function to segment the microscopy images based on the CD41 channel. The features “fill holes” and “watershed” were applied to the binary images to ensure that platelets were detected as individualized ROIs. We then used the “analyze particles” function to create ROIs and measure PAFAH1B1 signal intensity within these ROIs using the mean fluorescence intensity (MFI) method. For that, we used the “Analyze” function to extract the mean gray value of each ROI and corrected it to the background mean gray value (average mean gray value of three ROIs in a non-fluorescent area). We analyzed 3 microscopy fields (captured from different slides) per animal and a total of 306 platelets in young WT (4 months old), 777 in old WT (18-19 months old) and 707 in APP-PS1 mice. Statistical analysis was performed using GraphPad Prism (Version 7.00) using the average corrected MFI per animal. Data were tested for normality using the Shapiro-Wilk normality test and comparison between groups was performed by one-way ANOVA with Dunn’s multiple comparisons test. Data are depicted as mean ± standard error of the mean (SEM).

# Supplementary Figures and Tables

## Supplementary Figures

**Supplementary Figure 1.** **Mouse platelets express PAFAH1B1 and PSMB8.** Mouse platelets in blood smears **(A)** and in platelet rich plasma **(B)** were immunostained for qualitative analysis of PAFAH1B1 (red) and PSMB8 (white) expression. CD41 (green) was used to visualize platelets, and DAPI (blue) was used as nucleus staining. Mouse platelets express PAFAH1B1 and PSMB8. Scale bars: 10 µm. **(C)** Platelets in platelet rich plasma of WT and APP-PS1 mice expressed similar levels of PAFAH1B1. Data are shown as mean ± SEM (n =4/group, all males). Statistical analysis was performed by one-way ANOVA with Dunn’s multiple comparisons test.


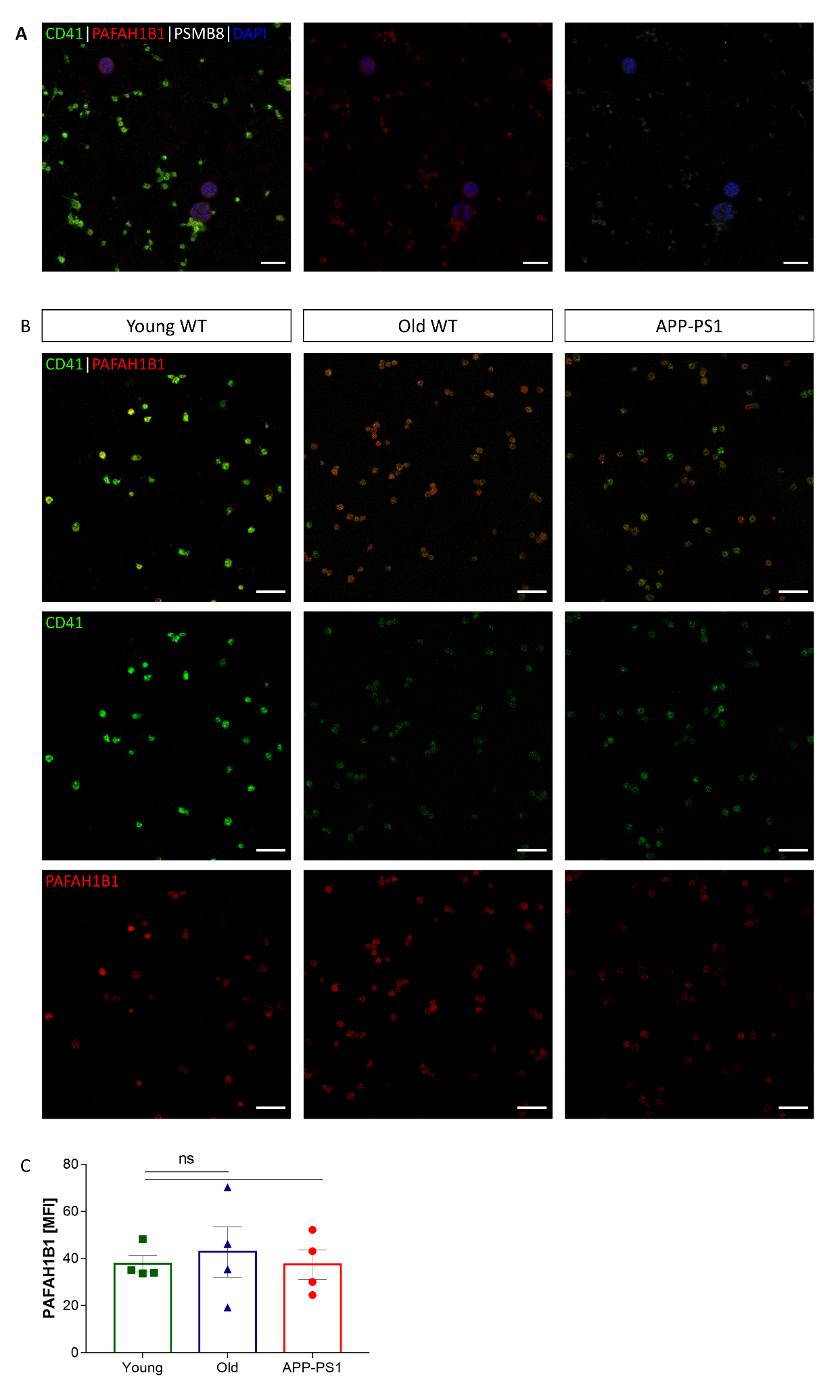


## Supplementary Tables

**Supplementary Table 1.** Exclusion criteria: Drugs with influence on platelet function

| **Pharmacological Class** | **Drugs** | **Washout Period** | **Exclusion** |
| --- | --- | --- | --- |
| **Platelet aggregation inhibitors** | *Aspirin* | 7 days | Yes, if the medication was taken during the washout period |
|  | **P2Y12 receptor ihnibitors** *Clopidogrel, Prasugrel, Ticagrelor, Ticlopidine* |  |  |
|  | **Phosphodiesterase inhibitors** *Dipyridamole, Cilostazol* | n.d. | Yes |
|  | **Glycoprotein IIb/IIIa receptor antagonist** Abciximab, Eptifibatide, Tirofiban |  |  |
| **Anticoagulants** | **Anticoagulants, oral** *Dabigatran, Phenprocoumon, Warfarin* | 7 days | Yes, if the medication was taken during the washout period |
|  | **Indirect factor Xa inhibitors** *Apixaban, Edoxaban, Rivaroxaban, Betrixaban* |  |  |
|  | **Low molecular weight heparins** *Enoxaparin, Dalteparin, Tinzaparin, Certoparin, Bemiparin, Nadroparin* |  |  |
| **Non-steroidal anti-inflammatory drugs (NSAIDs)** | **Non-selective NSAIDs** *Aspirin, Ibuprofen, Naproxen, Diclofenac, Indomethacin, Meloxicam, Piroxicam, Mefenamic acid, and Lornoxicam, Propephenazone* | 7 days | Yes, if the medication was taken during the washout period |
|  | **COX-2 selective NSAIDs** *Celecoxib, Etoricoxib* |  |  |
| **Analgesic non-opidoid** | *Metamizol* | 7 days | Yes, if the medication was taken during the washout period |
| **β-lactam antibiotics** | Penicillins Cephalosporins | 3 days | Yes, if the medication was taken during the washout period |
| **Drugs for the treatment of bone diseases** | *Strontium ranelate* | 4 weeks | Yes, if the medication was taken during the washout period |
| **Selective Estrogen Receptor Modulators (SERMs)** | *Raloxifen* | 4 weeks | Yes, if the medication was taken during the washout period |
| **Selective serotonin reuptake inhibitors (SSRIs)** | Fluoxetine, Paroxetine, Sertraline | n.d. | Yes |

# Supplementary Data

**Supplementary Data 1.** Spreadsheet containing the lists of differentially expressed proteins per comparison, overlapping proteins with Geyer et al platelet proteomics dataset, and significantly enriched GO terms and pathways

**Supplementary Data 2.** Spreadsheet containing the lists of differentially expressed transcripts per comparison, overlapping transcripts with Supernat et al platelet transcriptomics dataset, and significantly enriched GO terms and pathways

**Supplementary Data 3.** Spreadsheet containing normalized DESeq2 counts from platelet RNAseq data

# References

Jankowsky, J. L., D. J. Fadale, J. Anderson, G. M. Xu, V. Gonzales, N. A. Jenkins, N. G. Copeland, M. K. Lee, L. H. Younkin, S. L. Wagner, S. G. Younkin and D. R. Borchelt (2004). “Mutant presenilins specifically elevate the levels of the 42 residue beta-amyloid peptide in vivo: evidence for augmentation of a 42-specific gamma secretase.” Hum Mol Genet **13**(2): 159-170.

Jankowsky, J. L., H. H. Slunt, T. Ratovitski, N. A. Jenkins, N. G. Copeland and D. R. Borchelt (2001). “Co-expression of multiple transgenes in mouse CNS: a comparison of strategies.” Biomol Eng **17**(6): 157-165.

Janota, C. S., D. Brites, C. A. Lemere and M. A. Brito (2015). “Glio-vascular changes during ageing in wild-type and Alzheimer’s disease-like APP/PS1 mice.” Brain research **1620**: 153-168.

Laboratory, T. J. “B6.Cg-Tg(APPswe,PSEN1dE9)85Dbo/Mmjax." 2022, from <https://www.jax.org/strain/005864>.

Lalonde, R., H. D. Kim, J. A. Maxwell and K. Fukuchi (2005). “Exploratory activity and spatial learning in 12-month-old APP(695)SWE/co+PS1/DeltaE9 mice with amyloid plaques.” Neurosci Lett **390**(2): 87-92.

Shihan, M. H., S. G. Novo, S. J. Le Marchand, Y. Wang and M. K. Duncan (2021). “A simple method for quantitating confocal fluorescent images.” Biochem Biophys Rep **25**: 100916.

Unger, M. S., E. Li, L. Scharnagl, R. Poupardin, B. Altendorfer, H. Mrowetz, B. Hutter-Paier, T. M. Weiger, M. T. Heneka, J. Attems and L. Aigner (2020). “CD8+ T-cells infiltrate Alzheimer’s disease brains and regulate neuronal- and synapse-related gene expression in APP-PS1 transgenic mice.” Brain, Behavior, and Immunity **89**: 67-86.

Unger, M. S., J. Marschallinger, J. Kaindl, C. Höfling, S. Rossner, M. T. Heneka, A. Van der Linden and L. Aigner (2016). “Early Changes in Hippocampal Neurogenesis in Transgenic Mouse Models for Alzheimer’s Disease.” Mol Neurobiol **53**(8): 5796-5806.

Unger, M. S., J. Marschallinger, J. Kaindl, B. Klein, M. Johnson, A. A. Khundakar, S. Roßner, M. T. Heneka, S. Couillard-Despres, E. Rockenstein, E. Masliah, J. Attems and L. Aigner (2018). “Doublecortin expression in CD8+ T-cells and microglia at sites of amyloid-β plaques: A potential role in shaping plaque pathology?” Alzheimer’s & Dementia **14**(8): 1022-1037.

Unger, M. S., P. Schernthaner, J. Marschallinger, H. Mrowetz and L. Aigner (2018). “Microglia prevent peripheral immune cell invasion and promote an anti-inflammatory environment in the brain of APP-PS1 transgenic mice.” Journal of neuroinflammation **15**(1): 274-274.
